# Comparison of Spinal Block Levels between Laboring and Nonlaboring Parturients Using Combined Spinal Epidural Technique with Intrathecal Plain Bupivacaine

**DOI:** 10.1155/2012/187132

**Published:** 2012-06-20

**Authors:** Yu-Ying Tang, Jie Zhou, Xiao-Hui Ren, Xue-Mei Lin

**Affiliations:** ^1^Department of Anesthesiology, West China Second Hospital, Sichuan University, Sichuan, Chengdu 610041, China; ^2^Department of Anesthesiology, Perioperative and Pain Medicine, Brigham and Women's Hospital, Harvard Medical School, 75 Francis Street, Boston, MA 02115, USA; ^3^Department of Anesthesiology, Median Area of Maternal and Child Care Service Center, Sichuan, Neijiang 641000, China

## Abstract

*Background*. It was suggested that labor may influence the spread of intrathecal bupivacaine using combined spinal epidural (CSE) technique. However, no previous studies investigated this proposition. We designed this study to investigate the spinal block characteristics of plain bupivacaine between nonlaboring and laboring parturients using CSE technique. *Methods*. Twenty-five nonlaboring (Group NL) and twenty-five laboring parturients (Group L) undergoing cesarean delivery were enrolled. Following identification of the epidural space at the L3-4 interspace, plain bupivacaine 10 mg was administered intrathecally using CSE technique. The level of sensory block, degree of motor block, and hemodynamic changes were assessed. *Results*. The baseline systolic blood pressure (SBP) and the maximal decrease of SBP in Group L were significantly higher than those in Group NL (*P* = 0.002 and *P* = 0.03, resp.). The median sensory level tested by cold stimulation was T6 for Group NL and T5 for Group L (*P* = 0.46). The median sensory level tested by pinprick was T7 for both groups (*P* = 0.35). The degree of motor block was comparable between the two groups (*P* = 0.85). *Conclusion*. We did not detect significant differences in the sensory block levels between laboring and nonlaboring parturients using CSE technique with intrathecal plain bupivacaine.

## 1. Introduction

Combined spinal epidural (CSE) anesthesia is commonly used for cesarean delivery. It has been suggested that nonlaboring parturients have a higher sensory block level than those in labor during CSE anesthesia [1]. This proposition was derived from combining two independent randomized studies on spinal block levels designed separately for laboring and nonlaboring parturients [2, 3]. There was a 5-dermatome level difference between nonlaboring (C6) [2] and laboring (T3) [3] parturients using 10 mg hyperbaric bupivacaine with CSE technique. However, there has been no previous study examining the effect of labor on the level of the subarachnoid block during CSE. The effect of CSE technique on the spinal block level of hyperbaric bupivacaine in nonlaboring parturients was not consistent. Horstman et al. reported that sensory block level was at T3 with CSE in nonlaboring parturients using 20% higher dose of hyperbaric bupivacaine [4]. We speculate that baricity of the hyperbaric bupivacaine used by Ithnin et al. could be a confounding factor, because the block level could be easily manipulated with the positioning of the parturients when hyperbaric local anesthetic was used. The effect of labor on the spread of local anesthetics may be better examined by using plain or isobaric agents which hold the least gravity-generated flow dynamics in cerebrospinal fluid (CSF). This study was therefore designed to compare the spinal block characteristics between the laboring and nonlaboring parturients using plain bupivacaine injected intrathecally with the needle-through-needle CSE technique.

## 2. Methods

This research was conducted at the West China Second Hospital of Sichuan University, Chengdu, Sichuan Province, China. With median two-segment dermatome blockade level difference to be clinically significant and variability (interquartile range) of two dermatomes in sensory block, 20 parturients in each group were required in each group to address a 2-segment difference with a power of 0.9 and a level of significance of 5%.

Following the institutional research ethics board (IRB) approval and written informed consents, we enrolled two groups of parturients with 25 in each group. Group NL were 25 nonlaboring parturients undergoing elective cesarean delivery, and Group L were 25 parturients in spontaneous active labor (having regular uterine contractions and cervical dilation greater than 3 cm) undergoing cesarean delivery for failure to progress. All parturients were between 20 and 40 years of age, ASA physical status I-II with a singleton pregnancy at greater than 36 weeks gestation, and received a lower segment transverse incision for cesarean delivery. The decision for cesarean delivery was made by the obstetric team, independent of the study investigators. Exclusion criteria included parturient refusal, having received any analgesic treatment, any contraindication to CSE or general anesthesia, height less than 150 cm or greater than 175 cm, body weight greater than 100 kg, obstetric and/or medical comorbidities such as preeclampsia, any cardiac, renal, neurologic, or other systemic diseases, unilateral block, or maximal pinprick sensory block level below T10 at 20 min postspinal injection.

Each parturient was preloaded with 500 mL of lactated Ringer's solution intravenously (IV). On arrival in the operation room, baseline vital signs were recorded and the initial severity of labor pain for a laboring parturient was assessed on a 10 cm visual analog scale (VAS) before CSE anesthesia was placed. The degree of cervical dilations for laboring parturient was also recorded.

One anesthesiologist performed all CSE procedures following previously published methodology [2, 3]. With the parturient in the right lateral decubitus position, epidural space was identified at the L3-4 interspace with a 17-gauge Tuohy needle using the loss of resistance to air technique. The L3-4 interspace was identified by the line connecting the iliac crests to cross the spine. The volume of air used was limited to no more than 2 mL. Using the needle-through-needle technique, a 25-gauge Whitacre spinal needle (BD Durasafe) was advanced via the epidural needle with the orifice facing cephalad direction. When cerebrospinal fluid was detected, 10 mg plain bupivacaine (2 mL of 0.5% w/v bupivacaine), which was the same as the previously published studies by Ithnin and Lim et al. [2, 3], was injected over 10 seconds without barbotage or aspiration. Immediately after intrathecal injection, the epidural needle was removed without inserting an epidural catheter. All parturients were promptly placed in a supine position with left uterine displacement.

A second anesthesiologist recorded vital signs at two-minute intervals for 20 minutes. IV phenylephrine 100 *μ*g was administrated for a decrease in systolic blood pressure (SBP) to less than 90 mmHg or a 30% reduction in SBP and was repeated as necessary. Bradycardia with less than 50 beats per minute was treated with IV atropine 0.3 mg. Sensory level of the block was assessed bilaterally at two-minute intervals using alcohol swab (sensory blockade level to cold ) and 25-gauge hypodermic needle (sensory blockade level to pinprick) in the cephalad direction. The degree of motor blockade was evaluated bilaterally using the modified Bromage scale [5]. The peak sensory and motor blockade and the amount of time taken to achieve it were compared. Other side effects such as shivering, nausea, and vomiting were recorded.

Skin incisions were made 20 minutes after intrathecal injection. Prior to delivery of the fetuses, any reaction to surgical stimulation (including any verbal complaint of pain or withdrawal of extremities to pinprick) was supplemented with IV ketamine at a dose of 0.5 mg/kg each time until adequate anesthesia was achieved. After childbirth, midazolam 0.03 mg/kg was administrated to all parturients, and IV pethidine 0.5 mg/kg each time was also utilized according to parturient's reaction to ensure adequate anesthesia. General anesthesia was reserved as a back-up plan. Time intervals from skin incision to uterine incision, from uterine incision to fetus delivery, and surgical time were collected. The Apgar scores and parturients' satisfaction with anesthesia care were recorded. All parturients were followed for three days postoperatively.

### 2.1. Statistical Analysis

Statistical analysis was performed using SPSS Version 13.0 software. One-sample Kolmogorov-Smirnov test was applied to analyze the distribution of the data. Student's unpaired *t*-test was used to examine the differences of quantitative data between groups. The Mann-Whitney *U* test was performed to analyze the ordered variables, such as median sensory levels, VAS scores, modified Bromage scores, and those skewed quantitative data. The incidences of hypotension, shivering, and nausea/vomiting between the two groups were compared with Fisher's exact test. The correlations between sensory blockade level and cervical dilation or labor pain scores were analyzed with Spearman's rank correlation. A *P* value <0.05 was considered statistically significant.

## 3. Results

All 50 parturients enrolled completed the study. No technical difficulty or inadvertent dural puncture was encountered. Demographical data including age, weight, height, and gestation weeks were presented in Table 1.

All parturients had similar bilateral sensory block level, and all the peak pinprick sensory block levels were above T10. Median peak dermatomal sensory block level to pinprick and cold sensation and the time taken to achieve it in 20 min after spinal injection were comparable between the two study groups (Table 2).

Spearman's correlation coefficients were 0.07 and −0.44, respectively, for sensory blockade level to pinprick in relation with cervical dilation and labor pain scores (*P* = 0.81 and *P* = 0.10, resp.). In addition, Spearman's correlation coefficients for sensory blockade level to cold in relation with cervical dilation and labor pain scores were 0.22 and 0.17, respectively (*P* = 0.43 and *P* = 0.54, resp.). During surgery, there were separately 14 nonlaboring parturients and 8 laboring parturients required rescue analgesic (*P* = 0.15). The median rescue ketamine dosage was 30 mg (IR: 0–50 mg) for nonlaboring parturients and 0 mg (IR: 0–40 mg) for laboring parturients (*P* = 0.21). The median rescue pethidine dosage was 30 mg (IR: 0–40 mg) for nonlaboring parturients and 0 mg (IR: 0–40 mg) for laboring parturients (*P* = 0.19). No conversion to general anesthesia was warranted.

With regard to hemodynamic variables, the baseline SBP and the maximal decrease of SBP significantly higherwereobserved in the laboring group (*P* = 0.002 and *P* = 0.03, resp., Tables 1 and 2). No difference was found in the incidence of hypotension or the phenylephrine dosage between the two groups (Table 2). None of the parturients developed significant bradycardia. All parturients were satisfied with the anesthesia care. No incidence of postdural puncture headache or other neurological complications was noted postoperatively.

## 4. Discussion

Ithnin et al. attributed the significantly higher block level in nonlaboring parturients with CSE technique to the neutralization of the negative pressure of the epidural space and reduction of the dural sac volume [2]. Researchers from the same institution also concluded that labor-induced variable increase of epidural pressure countered the above effects [3]. Combining the aforementioned two studies, Macarthur suggested that CSE might generate higher block level in nonlaboring parturients [1]. However, we demonstrated that there was no statistical difference in the level of sensory blockade to either pinprick or cold between laboring and nonlaboring parturients with CSE technique. Neither did the block level relate to cervical dilation nor did the severity of labor pain. Within the same line of out findings, Horstman et al. confirmed that, compared to simple spinal technique, the change of epidural pressure from CSE technique was negligible to cause significant change of intrathecal anesthetic spread [4]. We believe that with the relatively fixed size and low compliance nature of the skeletal spinal canal, physiological labor contraction would only generate hydraulic pressure evenly transmitted along the spinal dural sac, leaving little fluid dynamic alteration to the intrathecally administered local anesthetics. This was also confirmed by Dubelmanand Forbeswho found that vigorous coughing had no influence on the cephalad spread of spinal anesthesia [6].

With the same CSE technique, the median peak sensory level in the present study achieved was no higher than T5, while previous studies using same amount of hyperbaric bupivacaine reported a median peak sensory level between C6 [2] and T3 [3] on nonlaboring and laboring parturients, respectively. The only difference among the above three studies was the baricity of bupivacaine. It has been widely accepted that, under the influence of gravity, hyperbaric anesthetic solutions injected into spinal space at the L3-4 level (the peak of lumbar lordosis) are more likely to spread into and halt at the lower thoracic region (the nadir of thoracic kyphosis). Hirabayashi et al. demonstrated flattening of both lumbar lordosis and thoracic kyphosis during later stages of pregnancy using magnetic resonance imaging in Japanese women [7]. Interestingly, both the present study and the studies by Ithnin and Lim et al. were conducted on Asian parturients. We believe that the flattening of the curvatures of the spinal column, especially the disappearing of the upward slope in the high thoracic spine, could enhance the cephalad spread of intrathecally administered hyperbaric anesthesia solutions, leading to the unusually high blockade reported by Ithnin et al. Such effect was eliminated by using plain bupivacaine solution when block level was much less affected by curvatures of the spine and body position. We are not certain if these anatomic changes were specific to Asian parturients, as previous studies on western parturient showed that baricity had no differences in subarachnoid spread of bupivacaine, in which bupivacaine was used at higher dose or combined with opioids [8, 9].

Hypotension during spinal anesthesia primarily results from decreased systemic vascular resistance after blockade of preganglionic sympathetic fibers, and patients with a higher sympathetic drive are more prone to develop hypotension [10]. So, a laboring parturient with spinal anesthesia would be more prone to hypotension than a nonlaboring parturient because both epinephrine and norepinephrine levels are increased during labor [11]. In this study, we also found a greater decrease of SBP in laboring parturients. However, the compensation of the autologous transfusion with uterine contractions [12] and higher baseline SBP of laboring parturients resulted in similar incidence of hypotension or the phenylephrine dosage.

The choice of the dose of bupivacaine (10 mg) in the present study was based on previous studies [2, 3], which provided comparability among the studies. The median sensory blockade to pinprick was lagged at T7 in this study, which could not provide an absolute pain-free situation for parturients during cesarean operation. However, the inadequate blockade level was easily rectified by ketamine and pethidine as stated in the materials section; no significant adverse effect on the clinical outcome of parturient and neonate was noticed. Followups of parturients were satisfactory.

Selection bias was one of the concerns that could not be excluded completely from this study because of the non-randomized design. Laboring parturients were shorter in height (2.7 cm difference in average) and lighter in weight (3.6 kg difference in average) than those of nonlaboring parturients, which might in part explain the reason for their failure to progress resulting in cesarean delivery. However, it has been demonstrated that height, weight, and bodymass index did not influence the spread of sensory blockade after subarachnoidal injection of bupivacaine [13, 14]. The obese and nonobese parturients also had a similar effective dose of intrathecal bupivacaine for cesarean delivery [15]. Of course, a carefully designed randomized controlled trial would eliminate these possible confounding factors. Although the gestational age had a significant difference between two groups, all parturients we enrolled had pregnancy greater than 36 weeks. To avoid the potential interference in judgment of level of sensory loss, laboring parturients who had received analgesics prior to cesarean delivery were excluded from this study.

In conclusion, our study could not demonstrate difference in the block level of spinal anesthesia between the laboring and nonlaboring parturients using plain bupivacaine injected with the CSE technique.

## Figures and Tables

**Table 1:** Demographical data.

Demographical data	Group NL	Group L	*P* value
	(*n* = 25)	(*n* = 25)	
Age (years old)	30.3 (5.1)	29.7 (5.2)	0.52
Weight (kg)	68.3 (8.3)	64.7 (4.5)	0.07
Height (cm)	158.6 (5.1)	155.9 (4.5)	0.05
Gestational age (wk)	38.4 (1.3)	37.4 (1.4)	0.02
Initial cervical dilatation (cm)	0	6.7 (2.2)	<0.001
Initial VAS score of labor pain	0	7 (6–8)	<0.001
Baseline SBP (mmHg)	116.6 (11.5)	131.8 (6.1)	0.002
Time period from skin incision to uterine incision (minutes)	4.9 (1.5)	5.4 (1.1)	0.13
Time period from uterine incision to delivery (seconds)	57.3 (9.2)	56.8 (13.0)	0.06
Duration of operation (minutes)	48.0 (9.5)	52.3 (10.0)	0.54
Apgar score at 1 minute	10 (9-10)	10 (8–10)	0.32
Apgar score at 5 minutes	10 (10-10)	10 (9-10)	0.20

Results are expressed as the mean (SD) or the median (interquartile range), as appropriate.

**Table 2:** Block characteristics 20 minutes after intrathecal injection.

Block characteristics	Group NL	Group L	*P* value
	(*n* = 25)	(*n* = 25)	
Median sensory blockade level to cold	T6 (T4–T6)^a^	T5 (T4–T6)^a^	0.46
Time to achieve cold blockade level (minutes)	10.9 (2.1)^b^	10.7 (2.3)^b^	0.83
Median sensory blockade level to pinprick	T7 (T6–T8)^a^	T7 (T7-T8)^a^	0.35
Time to achieve pinprick blockade level (minutes)	11.2 (2.2)^b^	10.5 (2.6)^b^	0.36
Motor block grade	3 (3-4)^a^	3 (3-4)^a^	0.85
Time to achieve motor block (minutes)	8.0 (2.5)^b^	7.7 (2.6)^b^	0.42
Phenylephrine (*μ*g)	0 (0–100)^a^	0 (0-0)^a^	>0.99
Maximal decrease of SBP (mmHg)	23.2 (13.2)^b^	34.6 (9.2)^b^	0.03
Hypotension	8 (32%)^c^	5 (20%)^c^	0.52
Shivering	5 (20%)^c^	12 (48%)^c^	0.07
Nausea and vomiting	3 (12%)^c^	1 (4%)^c^	0.61

^a^ Results are expressed as the median (interquartile range); ^b^results are expressed as the mean (SD); ^c^results are expressed as the number of parturients (percent).
